# FOXE1 represses cell proliferation and Warburg effect by inhibiting HK2 in colorectal cancer

**DOI:** 10.1186/s12964-019-0502-8

**Published:** 2020-01-09

**Authors:** Weixing Dai, Xianke Meng, Shaobo Mo, Wenqiang Xiang, Ye Xu, Long Zhang, Renjie Wang, Qingguo Li, Guoxiang Cai

**Affiliations:** 1 Department of Colorectal Surgery, Fudan University Shanghai Cancer Center, 270 Dong’an Road, Shanghai, 200032 China; 2 Department of Oncology, Shanghai Medical College, Fudan University, 270 Dong’an Road, Shanghai, 200032 China; 3 Shanghai Jiaotong Univeristy Affiliated Sixth People’s Hospital, Shanghai, 200233 China; 4 Cancer institute, Fudan University Shanghai Cancer Center, Shanghai, 200032 China

**Keywords:** FOXE1, HK2, Glycolysis, Cell proliferation

## Abstract

**Background:**

Low expression of FOXE1, a member of Forkhead box (FOX) transcription factor family that plays vital roles in cancers, contributes to poor prognosis of colorectal cancer (CRC) patients. However, the underlying mechanism remains unclear.

**Materials and methods:**

The effects of FOXE1 on the growth of colon cancer cells and the expression of glycolytic enzymes were investigated in vitro and in vivo. Molecular biological experiments were used to reveal the underlying mechanisms of altered aerobic glycolysis. CRC tissue specimens were used to determine the clinical association of ectopic metabolism caused by dysregulated FOXE1.

**Results:**

FOXE1 is highly expressed in normal colon tissues compared with cancer tissues and low expression of FOXE1 is significantly associated with poor prognosis of CRC patients. Silencing FOXE1 in CRC cell lines dramatically enhanced cell proliferation and colony formation and promoted glucose consumption and lactate production, while enforced expression of FOXE1 manifested the opposite effects. Mechanistically, FOXE1 bound directly to the promoter region of HK2 and negatively regulated its transcription. Furthermore, the expression of FOXE1 in CRC tissues was negatively correlated with that of HK2.

**Conclusion:**

FOXE1 functions as a critical tumor suppressor in regulating tumor growth and glycolysis via suppressing HK2 in CRC.

## Mini abstract

Collectively, our results established FOXE1 as a critical tumor suppressor, regulating CRC cell growth and aerobic glycolysis through FOXE1/HK2 axis, which therefore could be a promising therapeutic target for CRC.

## Introduction

Colorectal cancer (CRC) is one of the most common malignant cancers worldwide [1]. Currently, tumor-node-metastasis (TNM) staging is the most widely accepted system for risk stratification in colorectal cancer [2]. Once patients are diagnosed with metastatic CRC, the prognosis would decrease strikingly [3]. Thus, identifying the underlying mechanisms and biomarkers for CRC progression is urgently warranted to facilitate early diagnosis and treatment of CRC.

Cancers share a common phenotype of uncontrolled cell proliferation and must efficiently generate the energy and macromolecules required for cellular growth [4, 5]. Thus, cancer cells exhibit enhanced metabolic dependence that distinguishes them from normal cellular counterparts in which they display augmented nutrient acquisition strategies coupled with increased flux through downstream anabolic pathways. Metabolic reprogramming during tumorigenesis is an essential process in nearly all cancer cells [6]. The Warburg effect is the first example of metabolic reprogramming that Otto Warburg discovered in 1920s [7]. Cancer cells prefer glycolysis to mitochondrial oxidative phosphorylation to generate adenosine triphosphate (ATP), regardless of the availability of oxygen. Many studies have confirmed that oncogenes and tumor suppressors, such as hypoxia-inducible factor-1a, Myc, p53, PTEN, and Ras can reprogram energy metabolism in cancer cells [8–11]. However, the mechanisms accounting for the activation of the Warburg effect and progression of CRC remains blurry.

The Forkhead box (FOX) transcription factor family is defined by a highly conserved winged helix DNA-binding domain and participates in a variety of biological processes including cell cycle, proliferation, invasion, and metastasis [12–15]. Also, some of these transcription factors play fundamental roles in regulating Warburg effect [16, 17]. FOXE1, an important member of FOX transcription factor family, has been proved in previous studies to be a transcriptional repressor. Recently, its expression was found to be significantly lower in cancer tissues than in paired normal tissues and silencing of FOXE1 contributed to poor prognosis for CRC patients [18]. Although the prognostic value of FOXE1 has been suggested in CRC, it is necessary to understand the exact roles of FOXE1 in the development and progression of CRC. To date, the functions and downstream signaling cascades of FOXE1 in CRC remain unclear and no previous studies have been conducted to explore the regulating effect of FOXE1 on aerobic glycolysis in CRC.

In this study, we investigated whether and how FOXE1 modulated glycolysis in CRC cells. We demonstrated here that FOXE1 repressed Warburg effect by inhibiting the expression of the glycolytic enzyme hexokinase 2 (HK2), a key mediator of aerobic glycolysis, in CRC cells. FOXE1 bound directly to the promoter region of HK2 and negatively regulated its transcription and thus prohibiting cell proliferation. These findings revealed a previously unrecognized mechanism of FOXE1 in human CRC by modulating the aerobic glycolysis and cell growth through regulation of HK2.

## Patients and methods

### CRC patient information

Tissue microarray (TMA) was constructed using 276 primary CRC tissue specimens as described previously [19]. All patients underwent curative surgery without any preoperative cancer treatment and followed up for at least 5 years or until death. Overall survival (OS) was defined as the time from surgery to death caused by any reasons. Disease-free survival (DFS) was defined as the time from primary surgery to any local or distant relapse or end of follow-up without any relapse. Written informed consents were obtained from the patients and this study received approvals from the ethics committee of Fudan University Shanghai Cancer Center.

### Tissue preparation and immunostaining

Immunostaining of mouse tumor and TMA sections was conducted as previously described [19, 20] These antibodies were used: goat polyclonal anti-FOXE1 (ab5080, 1:100; Abcam) and rabbit polyclonal anti-HK2 (ab104836; 1:200; Abcam).

### Human CRC cell lines

NCM460, HT29, SW620, SW480, HCT116, and LoVo human CRC cell lines were obtained from the American Type Culture Collection (ATCC), which performed characterization or authentication of the cell lines using short tandem repeat profiling, regularly tested for mycoplasma contamination by using PCR and Hoechst staining.

### Expression vectors and gene transfection

Full-length FOXE1 and HK2 cDNAs were cloned into pCDH-CMV-MCS-EF1-Puro vector to generate pCDH-FOXE1 and pCDH-HK2 overexpression plasmids, respectively. Short hairpin RNA (shRNA) constructs against FOXE1 and HK2 was generated using pLKO.1-TRC cloning vector (Addgene, #10878). The shRNA target sequences for FOXE1 were 5′-CGTGGAGACCACGGTGGACTT-3′(sh1#) and 5′- CCCTCCACCTACCCGGCTTA-3′(sh2#). The target sequences for HK2 were 5′-ACTGAGTTTGACCAGGAGATT-3′(sh1#) and 5′-CACTGTGAAGTTGGCCTCA TT-3′(sh2#). Each constructed plasmid was co-transfected with the packaging plasmids psPAX2 and PMD2.G into HEK293T cells using Lipofectamine 3000 reagent (Thermo Fisher Scientific) according to the manufacturer’s protocol. Virus particles were harvested 48 h after transfection. Transfection of the target cells with lentiviral particles were performed using polybrene (2 μg /mL, Sigma-Aldrich) pretreatment, and positive cells were selected with puromycin (2 μg/mL, Sigma-Aldrich).

### Western blotting

Western blotting was performed using whole-cell protein lysates of CRC cells using primary antibodies against FOXE1 (ab236661, 1:1000; Abcam) and HK2 (ab37593,

1:1000; Abcam) and a secondary antibody (anti-rabbit IgG, 1:7500; Cell Signaling Technology). Equal loading of protein samples was monitored using an anti-β-actin antibody (ab8226, 1:2500; Abcam).

### RNA isolation and quantitative real-time reverse transcription polymerase chain reaction (qRT-PCR)

TRIzol reagent (Invitrogen, Carlsbad, CA, USA) was used to isolate total RNA and PrimeScript RT reagent (TaKaRa, Dalian, China) was used to obtain samples. The expression status of specific genes and β-actin were determined by qRT-PCR using an ABI 7900HT Real-Time PCR System (Applied Biosystems, Frederick, MD, USA). All reactions were run in triplicate.

### Cell apoptosis measurement

According to the manufacturer’s instructions, FITC Annexin V Apoptosis Detection Kit (BD, La Jolla, CA, USA) was used to detect apoptotic rate of cells.

### Cell viability and colony formation assay

Cell viability was measured by CCK-8 assay. For colony formation, cells were seeded into a six-well culture plates at a density of 500 cells/well and allowed to grow for 2 weeks. The cells were then fixed with methanol and stained with 0.1% crystal violet. All the visible colonies were counted manually.

### Lactate production and ATP level analysis

The cellular lactate production and ATP levels were measured using Fluorometric Lactate Assay Kit (Abcam) and Luminescent ATP Detection Assay Kit (Abcam) respectively.

### Glucose uptake assay

1 × 10^4^ cells were cultured in 96-well plates containing glucose-free DMEM (Thermo Fisher Scientific) with 10% fetal bovine serum (FBS, Thermo Fisher Scientific) and 6 mM glucose and then transferred to a CO_2_ incubator set at 37 °C and 5% CO_2_ for 48 h. Spent media were collected to measure remaining fructose using a glucose colorimetric/fluorometric assay kit (Abcam) following the manufacturer’s instruction.

### Extracellular acidification rate (ECAR) and oxygen consumption rate (OCR)

Seahorse Bioscience XF96 Extracellular Flux Analyzer was used to measure cellular mitochondrial function and glycolytic rate, following the manufacturer’s protocol of Seahorse XF Cell Mito Stress Test Kit or Glycolysis Stress Test Kit (Seahorse Bioscience, Billerica, MA, USA). Cells were plated in XF96 Cell Culture Microplates (Seahorse Bioscience) at a density of 4 × 10^4^ cells/well the day before measurement. Seahorse buffer consists of DMEM, phenolred, 25 mM glucose, 2 mM sodium pyruvate, and 2 mM glutamine. For ECAR measurement, 10 mM glucose, 1 μM oligomycin, and 100 mM 2-deoxy-glucose (2-DG) were automatically added to measure ECAR value. After monitoring baseline respiration, 1 μM oligomycin, 1 μM FCCP, and 1 μM rotenone were automatically injected into XF96 Cell Culture Microplates to measure the OCR. The ECAR and OCR values were calculated after normalization of cell number.

### Transcription activity analysis of HK2 promoter

The entire promoter region of HK2 was cloned and inserted into the luciferase promoter reporter vector, pGL3-Basic. The impact of FOXE1 on the transcriptional activity of the HK2 was assessed in 293FT and HCT116 cells by co-transfecting FOXE1, luciferase promoter reporter vectors containing HK2 promoter sequences, and Renilla luciferase reporter vector pRL-SV40 (Promega). The luciferase activity was detected by using the Dual-Luciferase Reporter System (Promega).

### Chromatin immunoprecipitation (ChIP) assay

ChIP assay to assess the binding status of FOXE1 with HK2 promoter was performed according to the standard manuals provided by Cell Signaling Technology Chromatin Immunoprecipitation Kit (Cell Signaling Technology). The resulted DNA samples were analyzed using PCR for the potential binding sites. The primer sequences was 5- GTGATATGCTAGTCACTTCAG − 3′ (sense) and 5′- TGCACGTCCTCAACCC TCCT − 3′ (antisense).

### Hexokinase activity assay

Extracting and assaying the hexokinase (HK) activity from CRC cells was performed according to the method developed by Christophe Ramière et al. [21] HK activity was measured spectrophotometrically through NADP+ reduction in a glucose-6-phosphate (Glc-6-P) dehydrogenase-coupled reaction.

### Mouse models and PET/CT analysis

We purchased 6–8 week-old female BALB/c-nude mice from Shanghai SLAC Laboratory Animal Co., Ltd. Mouse studies were performed in specific pathogen-free (SPF) facilities with the approval of the Institutional Animal Care and Use Committee of Fudan University. Subcutaneous xenograft mouse model was used in this study. All animal studies were conducted in accordance with the animal care guidelines at Fudan University Shanghai Cancer Center. For PET/CT study, the mice were starved for 8 h, then given 6 μCi ^18^F-FDG per gram body weight and undertook PET/CT scan 1 h later.

### Statistical analysis

All statistical analyses were performed using R software (R version 3.2.5, https://www.r-project.org/). Significant differences between two groups were computed using Wilcoxon rank-sum test for data with skewed distribution or Student’s test for data with normal distribution. Kaplan-Meier method and log-rank test were used to compare survival difference. Spearman rank correlation test was used to examine the association between FOXE1 and HK2 expression. *p* value < 0.05 was considered significant.

## Results

### Low FOXE1 expression is associated with poor prognosis of CRC

**Fig. 1:**
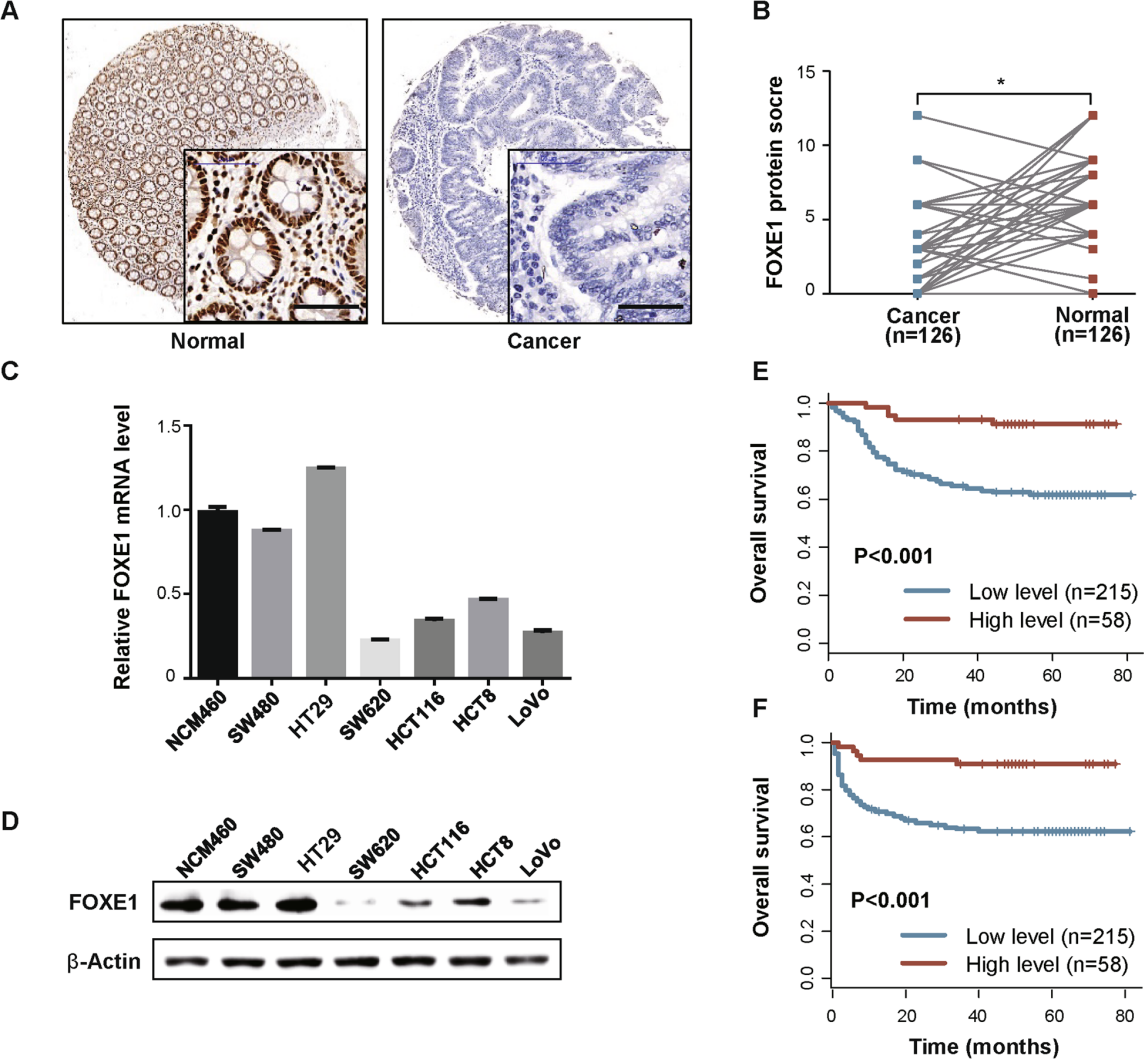
Low FOXE1 expression predicted poor survival for CRC. **a** Representative images showing low FOXE1 expression in CRC tissues (right panel) compared with adjacent normal tissues (left panel). **b** FOXE1 expression is significantly higher in paired normal tissues than in CRC tissue specimens (*P* < 0.001). **c** and **d** FOXE1 expression in one normal colonic epithelial cell NCM460 and six CRC cell lines determined using qRT-PCR analysis (**c**) and western blotting (**d**). **e** and **f** Kaplan–Meier analysis of the correlation of FOXE1 expression with OS (**e**) and DFS (**f**)

To investigate the prognostic value of FOXE1 in CRC cases, we tested its protein level in both CRC and paired normal tissues in TMA by IHC staining, which showed FOXE1 was highly expressed in normal mucosa compared with CRC tissues (Fig. 1a and b). In addition, in colon cancer cell lines, its low expression was detected inHCT116 and LoVoand high in SW480 and HT29(Fig. 1c and d). Correlation analysis showed that low expression of FOXE1 was significantly associated with poor clinicopathological features including advanced tumor stage and venous invasion (Additional file 3: Table S1). 17.9% of patents with low FOXE1 expression were diagnosed as metastatic CRC while only 5.2% of patents with high FOXE1 expression were stage IV disease (Additional file 3: Table S1). Further survival analysis suggested that FOXE1 expression was negatively associated with patients’ OS (*P* < 0.001) and DFS (*P* < 0.001) (Fig. 1e and f). These results demonstrated that FOXE1 may function as an important tumor suppressor in CRC progression and could be a vital biomarker for CRC prognosis.

### Enhanced FOXE1 expression inhibited cell growth in vitro and in vivo

**Fig. 2:**
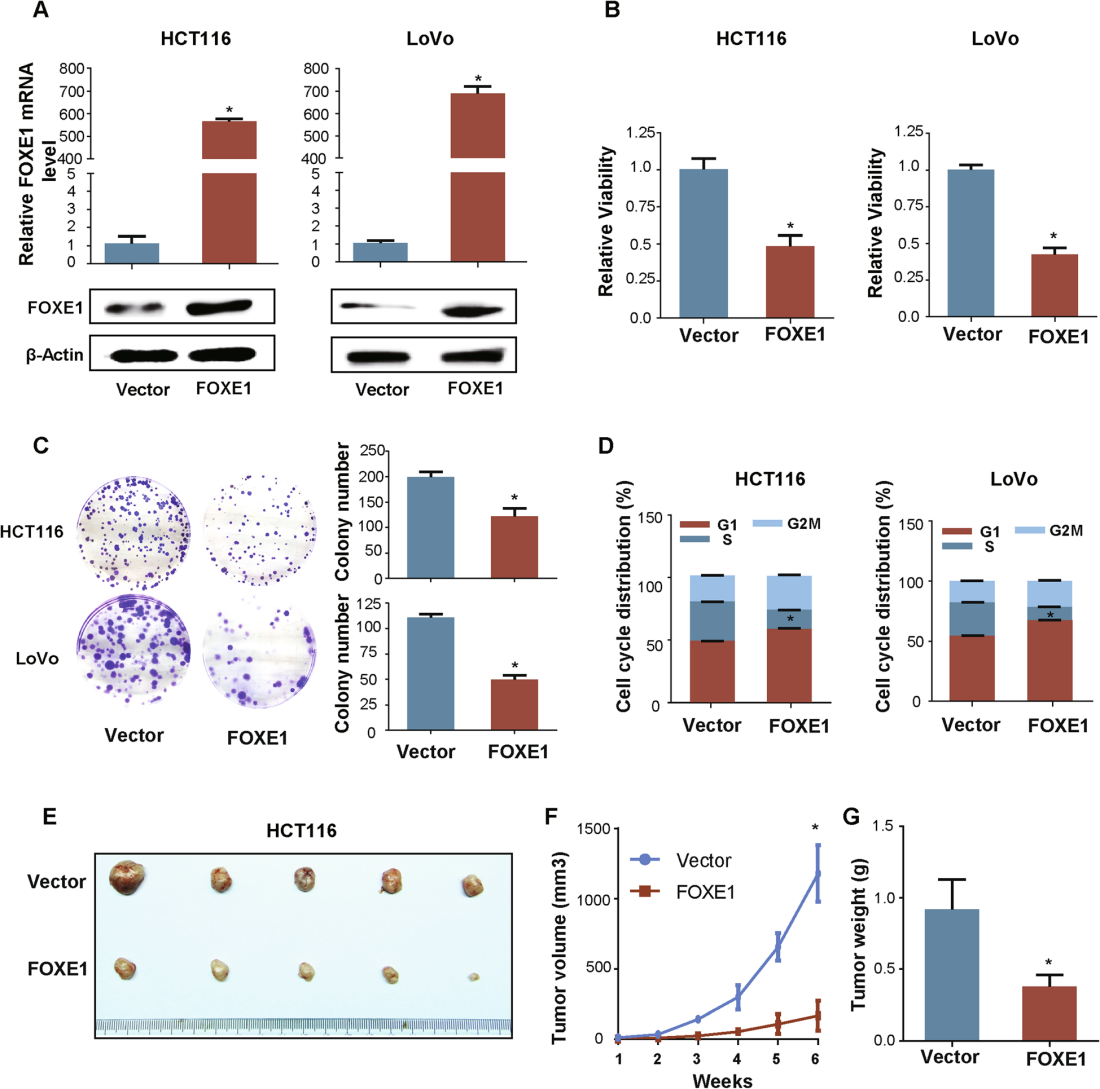
Enforced FOXE1 expression inhibited cell growth in vitro and in vivo. **a** Validation of over-expression FOXE1 in HCT116 and LoVo cells using western blotting and qRT-PCR. **b**, **c** and **d** The impact of FOXE1 expression on cell proliferation (**b**), colony formation (**c**) and cell cycle (**d**). **e**, **f** and **g** HCT116-Vector and HCT116-FOXE1 were subcutaneously injected into the right and left forelimb of five nude mice (5 × 10^6^ cells each xenograft). Gross xenografts (**e**), tumor growth curves (**f**) and tumors weights (**g**) are shown. *P < 0.05

To assess the role of FOXE1 in the proliferation of colon cancer cells, we overexpressed FOXE1 in HCT116 and LoVo cells. Western blotting and qRT-PCR were used to verify the overexpression of FOXE1 (Fig. 2a). In vitro, ectopic FOXE1 expression significantly suppressed cell viability (Fig. 2b), attenuated colony formation (Fig. 2c) and induced cell cycle arrest (Fig. 2d). Whereas, FOXE1 expression did not cause statistically significant changes in cell apoptosis (Additional file 1: Figure S1). Furthermore, the xenotransplant experiment showed that enforced FOXE1 expression significantly decreased the tumor-forming capacity of HCT116 cells (Fig. 2e-g).

### Silencing of FOXE1 promoted cell growth in vitro and in vivo

**Fig. 3:**
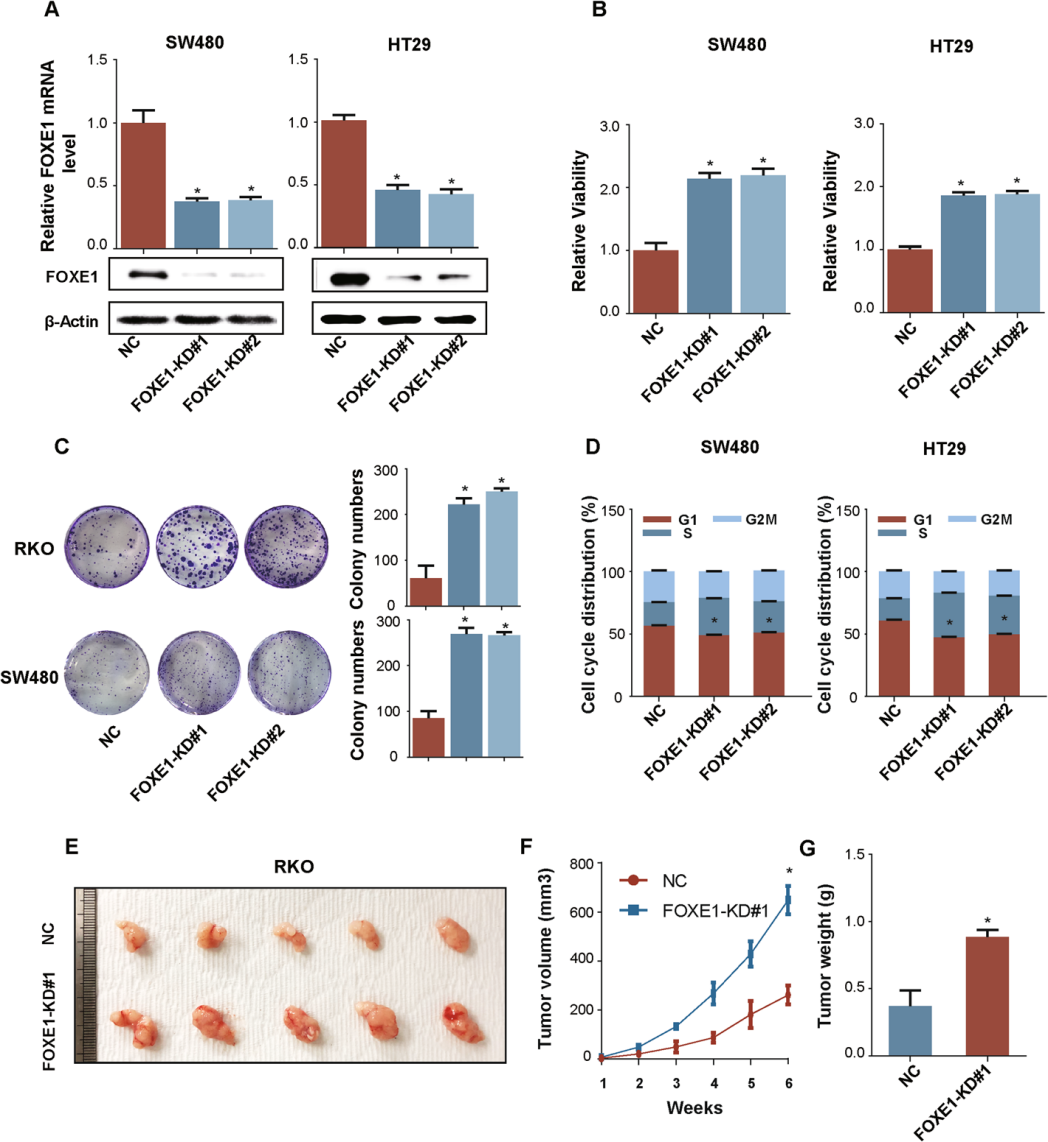
Silence of FOXE1 expression promotes cell growth in vitro and in vivo. **a** Validation of attenuated-expression FOXE1 in SW480 and HT29 using western blotting and qRT-PCR. **b**, **c** and **d** The impact of silenced FOXE1 expression on cell proliferation (**b**), colony formation (**c**) and cell cycle (**d**). **e**, **f** and **g**. SW480-NC and SW480-FOXE1-KD#1 were subcutaneously injected into the right and left forelimb of five nude mice (5 × 10^6^ cells each xenograft). Gross xenografts (**e**), tumor growth curves (**f**) and tumors weights (**g**) are shown. *P < 0.05

To further test whether attenuated FOXE1 expression could boost CRC cell growth, we silenced FOXE1 in SW480 and HT29 using shRNAs (Fig. 3a). In vitro, FOXE1 knockdown significantly enhanced cell proliferation and colony formation (Fig. 3b and c). Flow cytometry analysis showed that silencing of FOXE1 increased the S phase in cell cycle (Fig. 3d), but did not impact cell apoptosis (Additional file 1: Figure S1). In vivo study demonstrated that SW480 with silenced FOXE1 exhibited accelerated subcutaneous tumor growth (Fig. 3e-g).

### FOXE1 repressed glycolysis in CRC cells

**Fig. 4:**
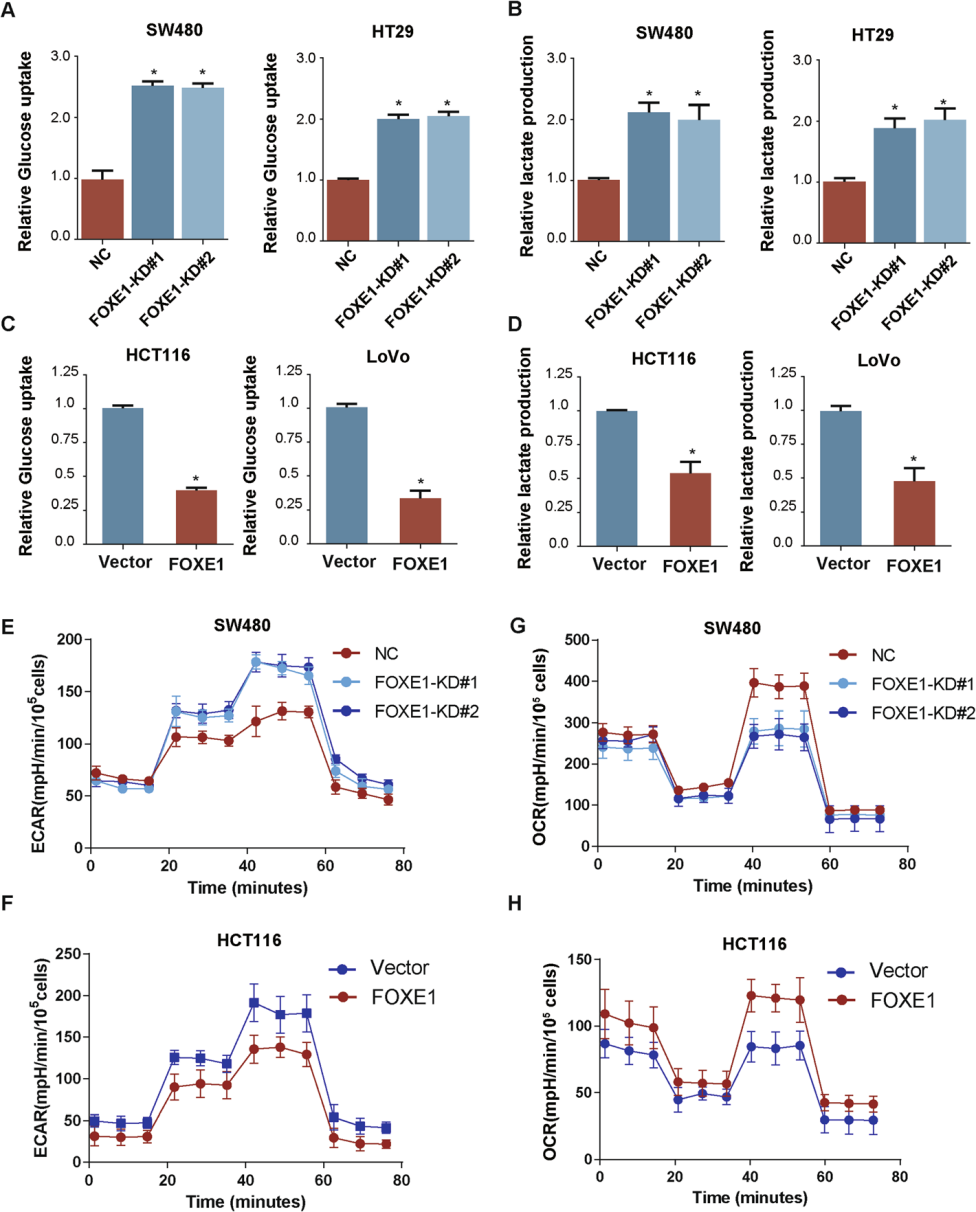
FOXE1 repressed glycolysis in CRC cells. **a**, **b** Attenuated FOXE1 expression promotes glucose uptake (**a**) and lactate proguction (**b**) in SW480 and HT29 cells. **c** and **d** Enforced expression of FOXE1 inhibits glucose uptake (**c**) and lactate proguction (**d**) in HCT116 and LoVo cells. **e** and **f** ECAR value increased significantly in FOXE1 silenced SW480 cell (**e**) but decreased in FOXE1 over-expressed HCT116 cell (**f**). **g** and **h** OCR value decreased significantly in FOXE1 silenced SW480 cell (**g**) but increased in FOXE1 over-expressed HCT116 cell (**h**). *P < 0.05

As a critical metabolic signature for invasive cancer, glycolysis plays an important role in the proliferation of CRC cells. Therefore, we investigated whether FOXE1 could modulate glycolysis in CRC cells to regulate their proliferation. Glycolysis analysis suggested that silencing of FXOE1 in HT29 and SW480 dramatically increased glucose consumption and lactate production (Fig. 4a and b), while ectopic expression of FOXE1 in HCT116 and LoVo cells reduced glucose uptake and lactate production significantly (Fig. 4c and d). We next used Seahorse XF Extracellular Flux Analyzers to examine the impact of FOXE1 on glycolysis, as reflected by ECAR. In FOXE1 silenced SW480 cells, the ECAR increased significantly (Fig. 4e). However, in FOXE1 overexpressed HCT116 cells, the ECAR decreased significantly (Fig. 4f). On the other hand, OCR results showed that OCR value deceased in FOXE1 silenced SW480 cells but (Fig. 4g) increased in FOXE1 overexpressed HCT116 cells (Fig. 4h).

**Fig. 5:**
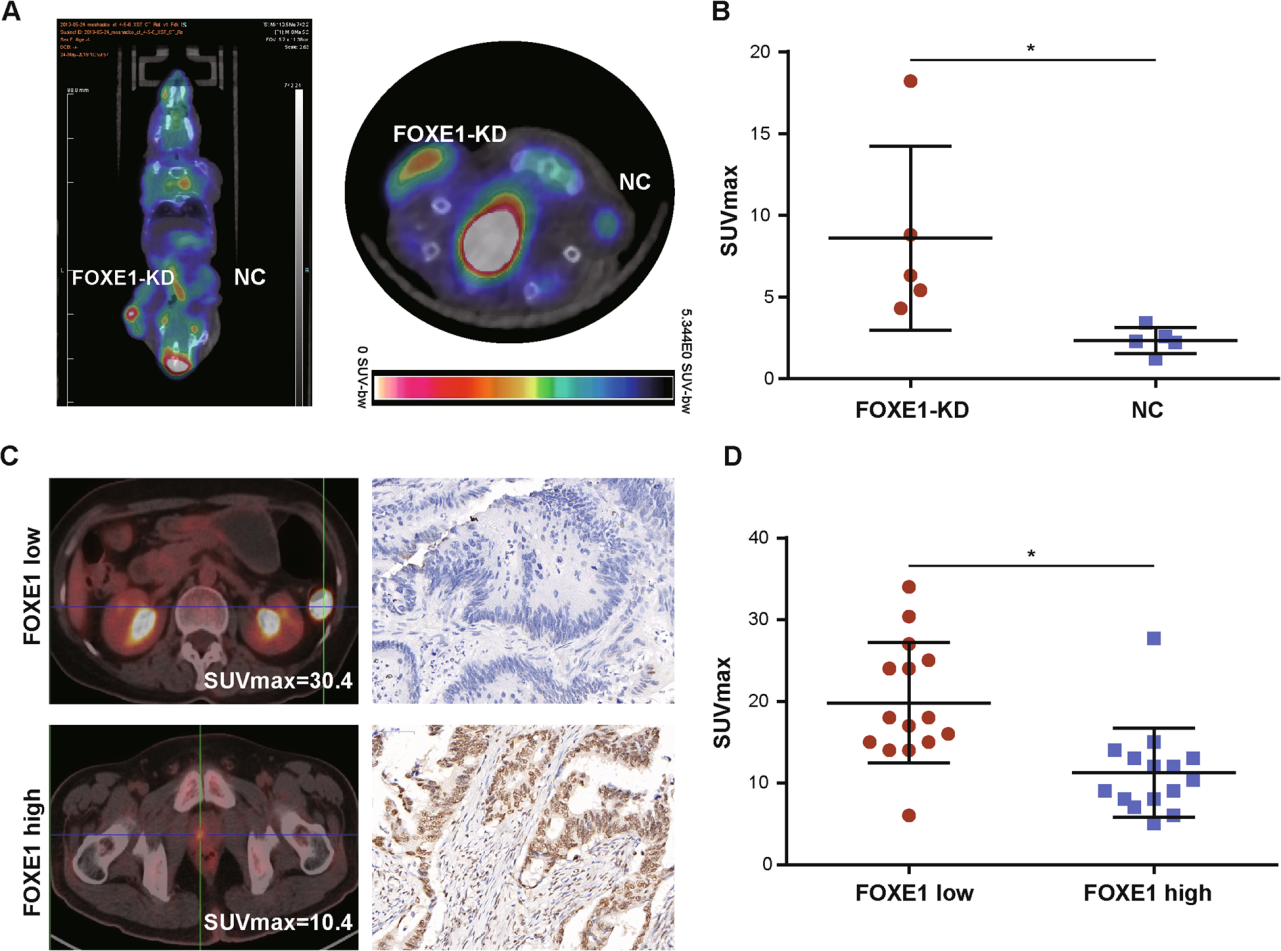
FOXE1 repressed glycolysis in vivo and FOXE1 expression is negatively correlated with18F-FDG PET/CT SUVmax value in CRC patients. **a** and **b** Representative photographs of ^18^F-FDG PET/CT scans of mice injected with HCT116-FOXE1-KD#1 and control(NC). The SUVmax was significantly higher in the HCT116-FOXE1-KD#1 group than in the control group (*P* < 0.05). **c** and **d** Representative photographs of ^18^F-FDG PET/CT scans and the corresponding FOXE1 IHC stains of CRC specimens. Patients with low expression of FOXE1 (red points) showed significantly higher SUVmax than patients high FOXE1 expression (blue points)

Based on Warburg effect, ^18^F-FDG positron emission tomography (PET) /computed tomography (CT) has been developed for clinical diagnosis of cancer. To investigate whether FOXE1 can impact glycolysis in vivo, we subjected SW480-FOXE1-KD (knockdown) and SW480-NC (negative control) injected mice to ^18^F-FDG PET/CT before sacrifice, which showed silencing of FOXE1 expression strikingly enhanced glycolysis as reflected by standard uptake value (SUVmax) (Fig. 5a and b). What’s more, in CRC patients who received preoperative ^18^F-FDG PET/CT examination, the SUVmax was significantly higher in the FOXE1 low expression group than the high expression group (Fig. 5c and d).

### HK2 is a transcriptional target of FOXE1 in CRC cells

**Fig. 6:**
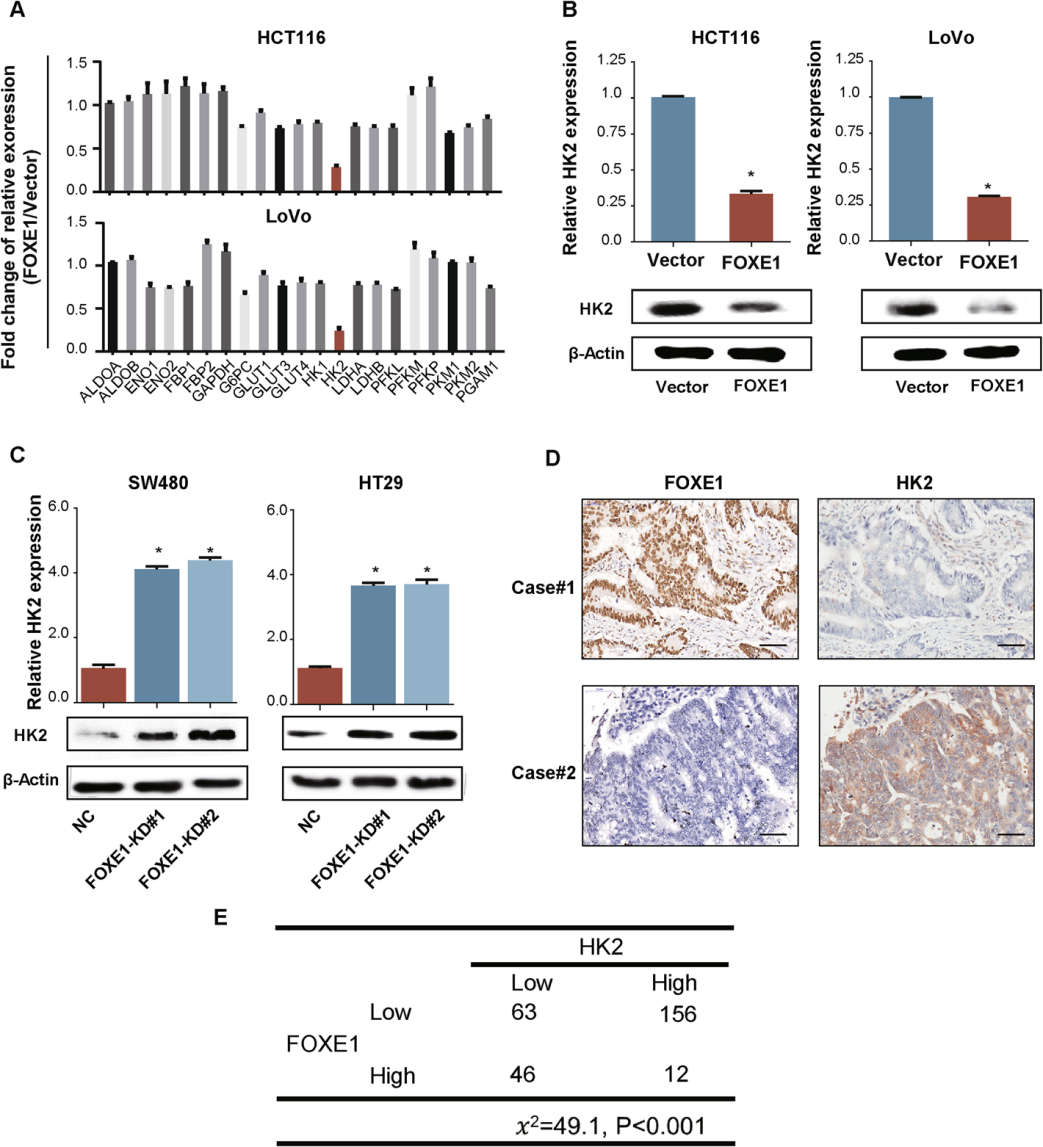
FOXE1 suppressed glycolysis via inhibiting HK2 expression. **a** Histograms showing the changes in mRNA expression for critical enzymes involved in glycolysis. **b** Forced FOXE1 expression inhibits the expression of the glycolytic enzyme HK2. **c** Attenuated FOXE1 expression promotes the expression of the HK2. **d**, **e** IHC stains of CRC specimens from patients showing that FOXE1 expression was negatively associated with HK2 expression. *P < 0.05

To investigate whether FOXE1 could repress glycolysis by regulating critical glycolytic enzymes, we performed qRT-PCR analysis to identify the glycolytic enzymes that might be regulated by FOXE1. We found that with enforced expression of FOXE1, among all the enzymes detected, only HK2 showed a significant decrease at the mRNA level (Fig. 6a and b). In addition, silencing FOXE1 in SW480 and HT29 cells significantly enforced HK2 expression (Fig. 6c). IHC was conducted using CRC TMA to validate the association between FOXE1 and HK2 from the protein level, which showed that FOXE1 expression was negatively correlated with HK2 expression (*P* < 0.05, Fig. 6d and e).

**Fig. 7:**
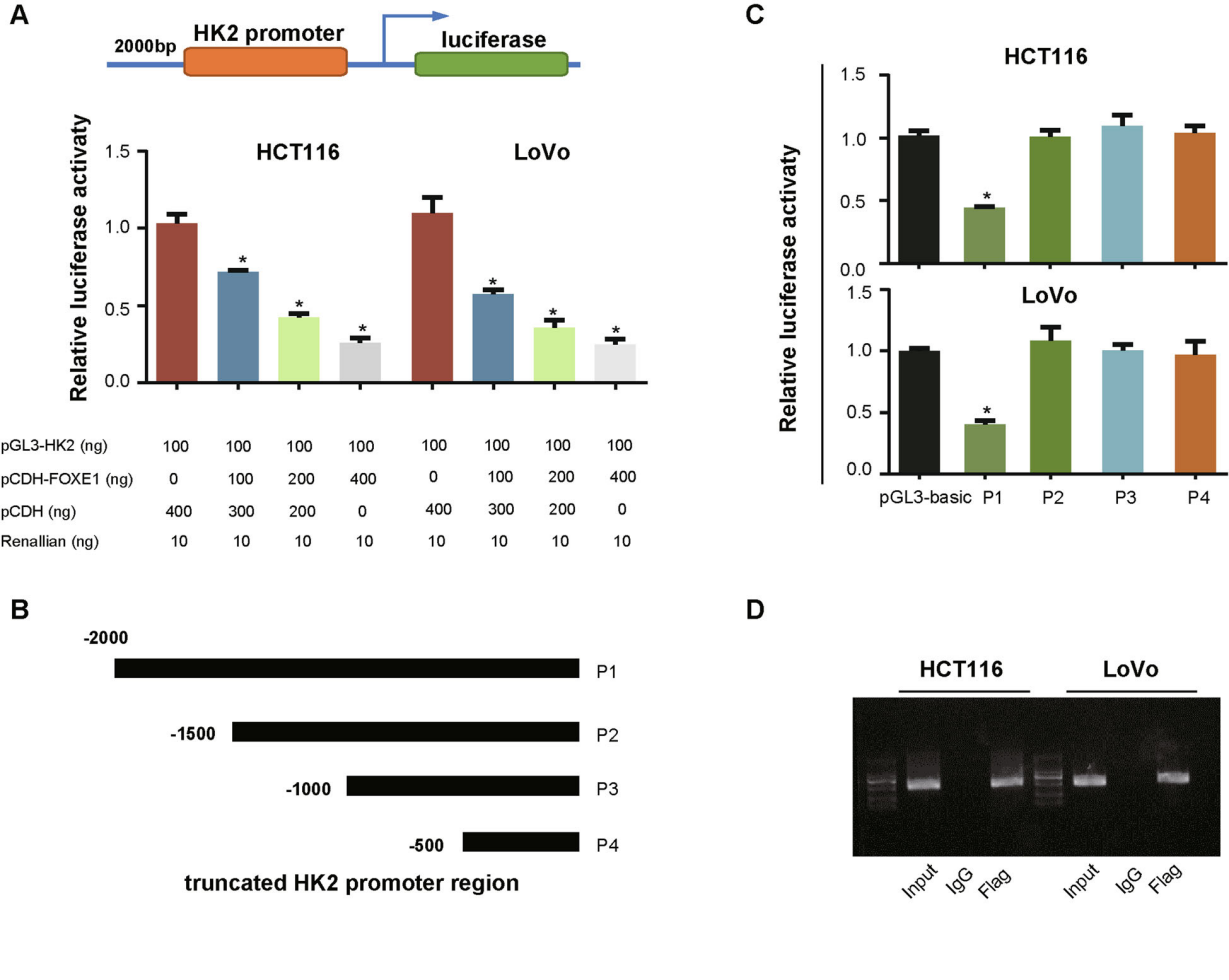
FOXE1 is a transcription factor of HK2. **a** Luciferase activity of the pGL3-HK2-Luc in HCT116 and LoVo cells being transfected with FOXE1 plasmid. **b** The truncated HK2 promoter regions were cloned into the pGL3 plasmid. **c** Enhanced FOXE1 expression strongly enhanced the promoter activity of the P1 but not the P2, P3 and P4 regions. **d** ChIP assays performed in HCT116 and LoVo cells. Specific anti-Flag antibody for ectopically expressed Flag-FOXE1, but not isotype IgG, captured the fragment that possibly containing the FOXE1 response element in the HK2 promoter region. *P < 0.05

To reveal whether FOXE1 regulates HK2 transcriptionally, dual luciferase assays were performed and the results indicated that enforced FOXE1 expression significantly reduced the luciferase activity of HK2 in CRC cells (Fig. 7a). To further confirm that FOXE1 can bound directly to the promoter regions of the HK2 gene, we next constructed a series of pGL3 plasmids containing 5’truncations of the HK2 promoter with different lengths (Fig. 7b). These plasmids were then co-transfected into CRC cells with the FOXE1-expressing plasmid or empty vector. The results of relative luciferase activity showed that ectopic FOXE1 expression significantly decreased transcriptional activity of the plasmids containing the P1 but not the P2, P3 and P4 HK2 promoter regions compared to the vector control (Fig. 7c), suggesting that the FOXE1 binding sites were probably located at the HK2 promoter region from -2000 bp to -1500 bp. ChIP assays demonstrated that FOXE1 could directly bound to the region from − 2000 to − 1500 bp of the HK2 (Fig. 7d). To further test whether HK2 enzymatic activity was regulated by FOXE1, Hexokinase activity assay was conducted and found that dysregulation of FOXE1 cannot influence the enzymatic activity (Additional file 2: Figure S2).

### HK2 enhanced glycolysis and cell growth in CRC

**Fig. 8:**
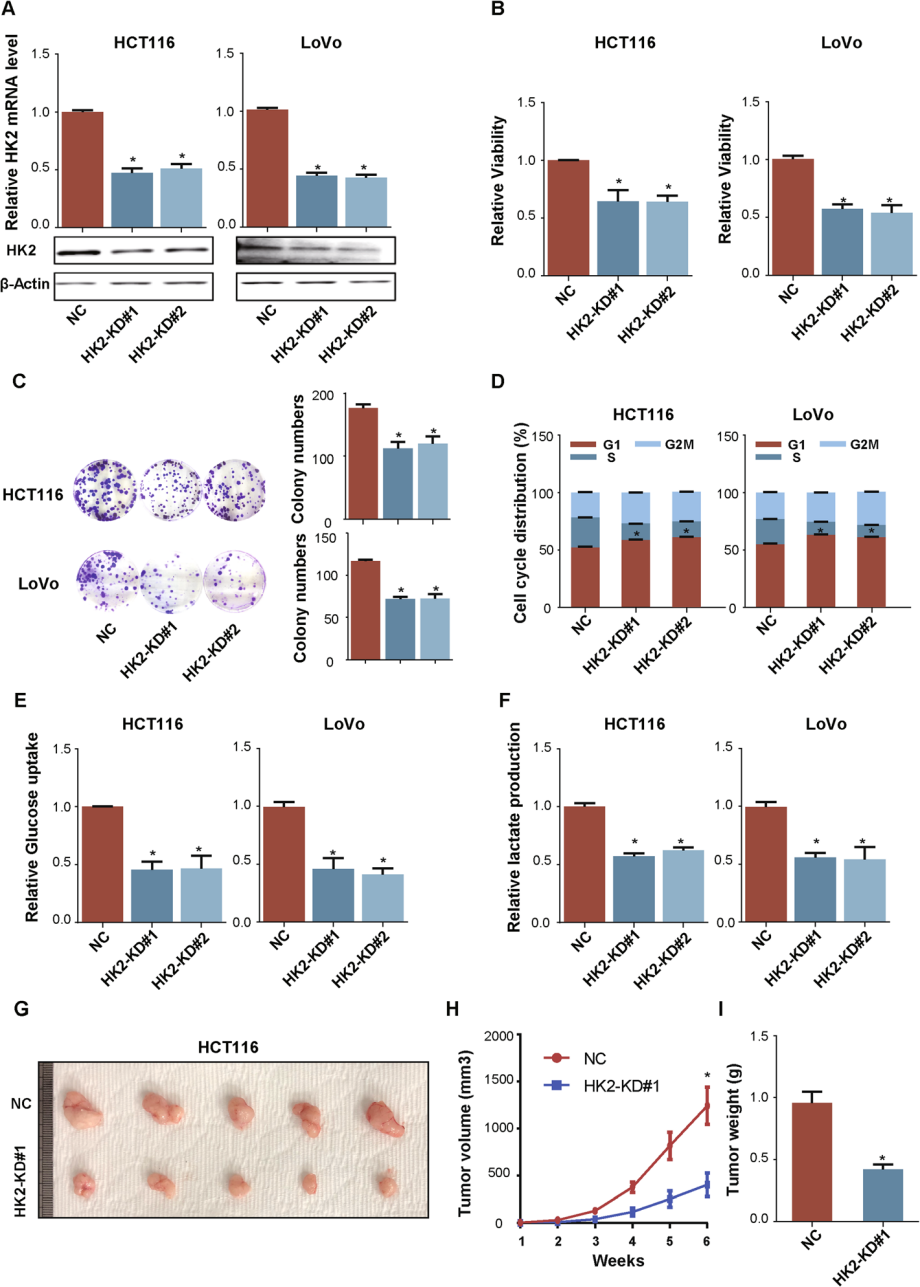
Silence of HK2 inhibited cell growth and glycolysis in CRC cells. **a** Validation of attenuated-expression HK2 in HCT116 and LoVo using western blotting and qRT-PCR. **b**, **c** and **d**. The impact of HK2 expression on cell proliferation (**b**), colony formation (**c**) and cell cycle (**d**). **e** and **f**. Silence of HK2 inhibited glucose uptake and lactate production. **g**, **h** and **i**. HCT116-NC and HCT116-FOXE1-KD#1 were subcutaneously injected into the right and left forelimb of five nude mice (5 × 10^6^ cells each xenograft). Gross xenografts (**g**), tumor growth curves (**h**) and tumors weights (**i**) are shown. *P < 0.05

To verify the roles of HK2 in promoting the glycolysis and proliferation of CRC cells, we silenced HK2 expression in HCT116 and LoVo cells (Fig. 8a) and found that cell growth (Fig. 8b) andcolony formation (Fig. 8c) were inhibited, and cell cycle arrest was induced(Fig. 8d). In addition, glucose uptake and lactate production were significantly decreased in cells with silenced HK2 (Fig. 8e and f). What’s more xenografts grew at a lower rate in mice injected with HCT116 cells expressing HK2 short hairpin RNA than that in the control group (Fig. 8g-i).

## Discussion

Our research focused on the effect of FOXE1 on CRC growth and glycolysis, which has not been studied before. We demonstrated here that FOXE1 is an important prognostic biomarker for CRC and its high expression can inhibit CRC growth and glycolysis in vitro and in vivo. Mechanistically, FOXE1 could down regulate the expression of glycolytic enzyme HK2 by negatively regulating its transcription.

In this study, FOXE1 prohibited the proliferation of CRC cells, providing new evidence for its role as a tumor suppressor in cancer development and progression. Previous studies have revealed that along with the rapid growth of solid tumors, it will be increasingly difficult for cancer cells to obtain sufficient oxygen and nutrient, thus contributing hypoxia and metabolic stress. The aerobic glycolysis promotes cancer cells to grow by providing both energy and biosynthesis building blocks and minimizing the reactive oxygen species generation in mitochondria [7]. Many tumor suppressors and oncogenes have been reported to influence cancer cell glycolysis by regulating the expression of glycolytic rate-limiting enzymes and specific glucose transporters [22]. For example, p53 suppresses glycolysis and tumor progression via downregulating the expression of phosphoglycerate mutase and glucose transporters 1 and 4 [23, 24]. Also, Akt activation because of PTEN loss contributed to the stabilization of glycolytic enzymes of phosphofructokinase 1 [25]. Activation of Myc could enhance glycolysis by upregulating lactate dehydrogenase A and phosphoinositide-dependent kinase 1 expression [26, 27].

Members of the FOX family are important transcriptional factors and are characterized by a distinct DNA-binding forkhead domain. FOX factors play a vital role in a variety of biological processes including energy homeostasis [15]. FOXM1, has been found to promote glycolysis and tumor progression by activating many enzymes and glucose transporters including lactate dehydrogenase A, HK2 and glucose transporter 1 [16, 28–30]. In the current study, we, for the first time, found that FOXE1 can repress glycolysis by down-regulating HK2. HK2 plays a vital role in aerobic glycolysis, catalyzing its first step and phosphorylating glucose to produce glucose-6-phosphate [31]. Previous studies have confirmed that the expression of HK2 is significantly upregulated in many cancers and its high expression in cancers is associated with poor prognosis [30, 32–35]. Administration of HK2 inhibitor, 2-DG, can induce cancer cell death by abrogating intracellular glycolysis. Therefore, HK2 is regarded as a key player in aerobic glycolysis and has been proposed as a therapeutic target for cancers [36].

Considering the fundamental roles of FOXE1 and HK2 in CRC progression and glycolysis, further experiments were conducted to explore the mechanism for the regulation of HK2 by FOXE1 in CRC cells. Since FOXE1 is a transcription factor, we used luciferase assays and confirmed that FOXE1 can attenuate the promoter activity of HK2. Of note, silencing HK2 expression in CRC cells has similar effect to FOXE1 overexpression. Therefore, we speculated that overexpression of HK2 in FOXE1 knockdown cells could reverse enforced cell growth and glycolysis elicited by silencing FOXE1.

Though our study revealed the significance of FOXE1 in CRC cell growth and glycolysis, the identification of specific transcription factor binding site on HK2 promoter for FOXE1 needs further experiment. To date, limited studies have focused on the biological process that FOXE1 participated in and no database can be used to predict the potential binding site of FOXE1 on the promoter region of target genes. Therefore, the specific and core DNA-binding sequence of FOXE1 needs to be determined.

## Conclusions

In conclusion, we, for the first time, investigated the function of FOXE1 in CRC cell growth and aerobic glycolysis and explored the potential molecular mechanism of FOXE1 in CRC malignancy maintenance. Our findings revealed FOXE1/HK2 is a novel regulatory axis modulating glycolysis and cell proliferation and is a promising therapeutic target for CRC.

## Supplementary information

**Additional file 1: Figure S1.** Altered expression of FOXE1 did not affect CRC cell apoptosis in vitro. A, B, C Impact of enforced FOXE1 expression on cell apoptosis in HCT116 and LoVo cells. D, E, F Impact of silenced FOXE1 expression on cell apoptosis in SW480 and HT29 cells. (n.s. no significance).

**Additional file 2: Figure S2.** FOXE1 expression did not influence HK activity. A, B Enhanced FOXE1 expression in HCT116 (A) and LoVo (B) cells did not impact HK activity. (n.s. no significance).

**Additional file 3: Table** **S1.** Comparison of baseline clinicopathological characteristics based on FOXE1 protein expression of CRC patients.

## Data Availability

Source data and reagents are available from the corresponding author upon reasonable request.

## Abbreviations

ATCC: American Type Culture Collection
ATP: Adenosine triphosphate
CRC: Colorectal cancer
DFS: Disease-free survival
ECAR: Extracellular acidification rate
FOX: Forkhead box
HK: Hexokinase
HK2: Hexokinase 2
OCR: Oxygen consumption rate
OS: Overall survival
qRT-PCR: quantitative real-time reverse transcription polymerase chain reaction
SPF: Specific pathogen-free
TMA: Tissue microarray
TNM: Tumor-node-metastasis
