# Supplementary figures and images for: FOXE1 represses cell proliferation and Warburg effect by inhibiting HK2 in colorectal cancer
Source: Cell Commun Signal. 2020 Jan 9;18:7. doi: 10.1186/s12964-019-0502-8 (PMC6953170; doi:10.1186/s12964-019-0502-8)

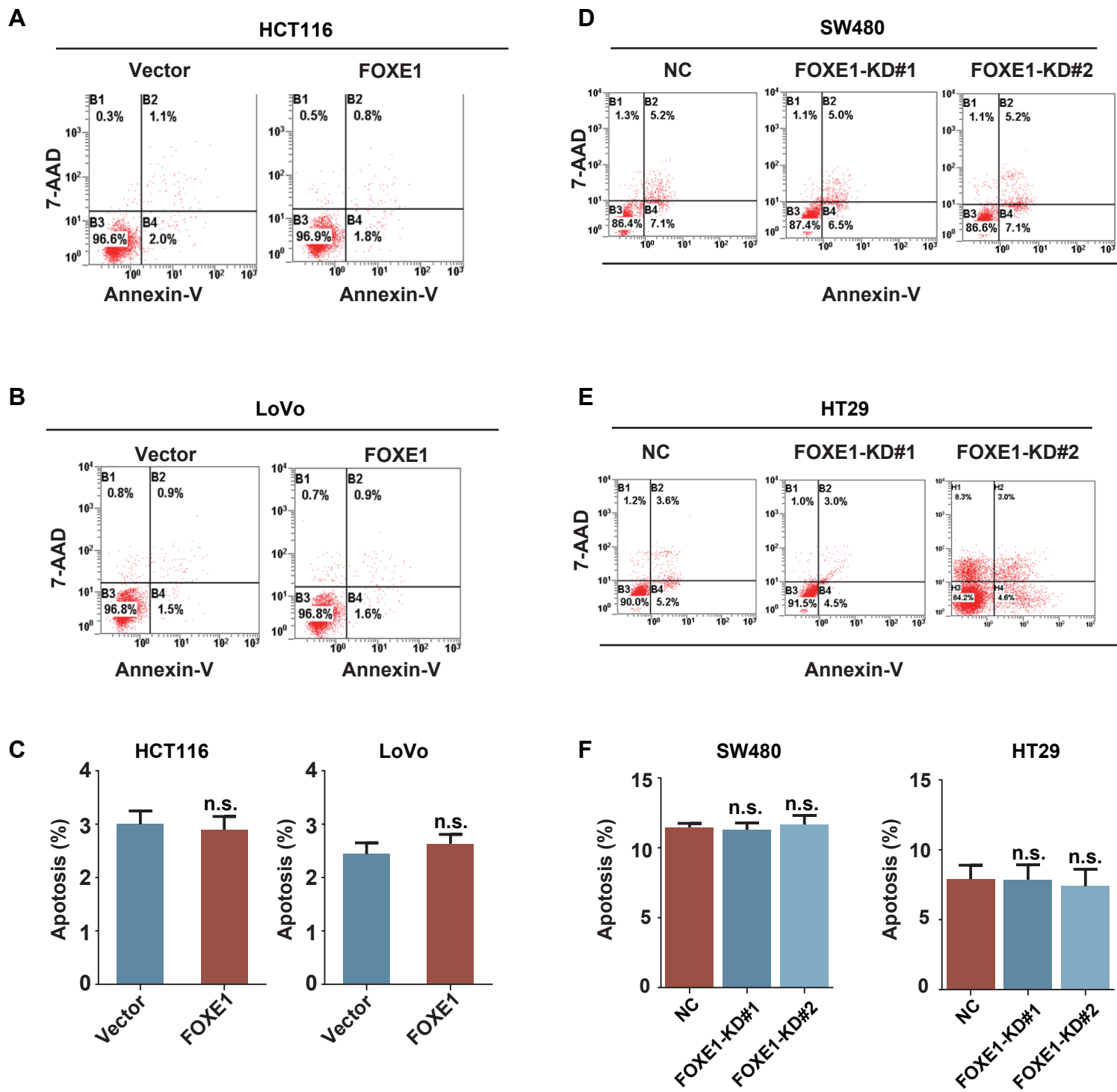

Figure S1

Supplement: Supplementary file 1 — Additional file 1: Figure S1. Altered expression of FOXE1 did not affect CRC cell apoptosis in vitro. A, B, C Impact of enforced FOXE1 expression on cell apoptosis in HCT116 and LoVo cells. D, E, F Impact of silenced FOXE1 expression on cell apoptosis in SW480 and HT29 cells. (n.s. no significance). [file 12964_2019_502_MOESM1_ESM.pdf]

**A**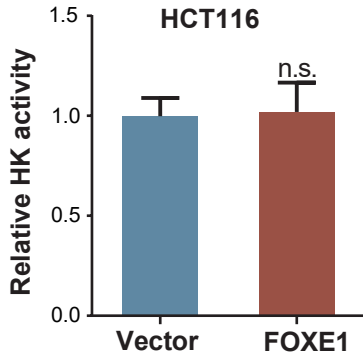**B**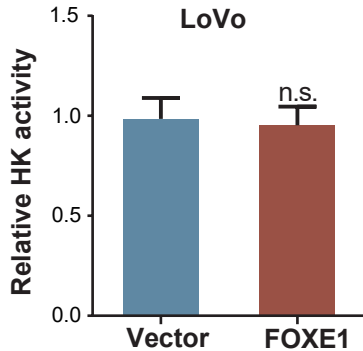

**Figure S2**

Supplement: Supplementary file 2 — Additional file 2: Figure S2. FOXE1 expression did not influence HK activity. A, B Enhanced FOXE1 expression in HCT116 (A) and LoVo (B) cells did not impact HK activity. (n.s. no significance). [file 12964_2019_502_MOESM2_ESM.pdf]
