# Supplementary material for: FOXE1 represses cell proliferation and Warburg effect by inhibiting HK2 in colorectal cancer
Source: Cell Commun Signal. 2020 Jan 9;18:7. doi: 10.1186/s12964-019-0502-8 (PMC6953170; doi:10.1186/s12964-019-0502-8)
Supplement: Supplementary file 3 — Additional file 3: Table S1. Comparison of baseline clinicopathological characteristics based on FOXE1 protein expression of CRC patients. [file 12964_2019_502_MOESM3_ESM.docx]

| **Table S1.** Comparison of baseline clinicopathological characteristics based on FOXE1 protein expression of CRC patients. | | | | | | | |
| --- | --- | --- | --- | --- | --- | --- | --- |
|  |  |  |  | FOXE1 | |  |  |
| Variables | Total | | Low level | | High level | | P |
|  | N | % | N | % | N | % |  |
| Age |  |  |  |  |  |  | 0.489 |
| ≤60 | 170 | 61.6% | 132 | 60.6% | 38 | 65.5% |  |
| >60 | 106 | 38.4% | 86 | 39.4% | 20 | 34.5% |  |
| Sex |  |  |  |  |  |  | 0.076 |
| male | 166 | 60.1% | 137 | 62.8% | 29 | 50.0% |  |
| female | 110 | 39.9% | 81 | 37.2% | 29 | 50.0% |  |
| Stage |  |  |  |  |  |  | <0.001 |
| I | 27 | 9.8% | 14 | 6.4% | 13 | 22.4% |  |
| II | 87 | 31.5% | 60 | 27.5% | 27 | 46.6% |  |
| III | 120 | 43.5% | 105 | 48.2% | 15 | 25.9% |  |
| IV | 42 | 15.2% | 39 | 17.9% | 3 | 5.2% |  |
| Tstage |  |  |  |  |  |  | 0.102 |
| T2 | 43 | 15.6% | 29 | 13.3% | 14 | 24.1% |  |
| T3 | 54 | 19.6% | 42 | 19.3% | 12 | 20.7% |  |
| T4 | 179 | 64.9% | 147 | 67.4% | 32 | 55.2% |  |
| Nstage |  |  |  |  |  |  | <0.001 |
| N0 | 120 | 43.5% | 80 | 36.7% | 40 | 69.0% |  |
| N1 | 82 | 29.7% | 72 | 33.0% | 10 | 17.2% |  |
| N2 | 74 | 26.8% | 66 | 30.3% | 8 | 13.8% |  |
| Mstage |  |  |  |  |  |  | 0.017 |
| M0 | 234 | 84.8% | 179 | 82.1% | 55 | 94.8% |  |
| M1 | 42 | 15.2% | 39 | 17.9% | 3 | 5.2% |  |
| Perineural invasion | |  |  |  |  |  | 0.855 |
| No | 231 | 83.7% | 182 | 83.5% | 49 | 84.5% |  |
| Yes | 45 | 16.3% | 36 | 16.5% | 9 | 15.5% |  |
| Venous invasion | |  |  |  |  |  | 0.007 |
| No | 188 | 68.1% | 140 | 64.2% | 48 | 82.8% |  |
| Yes | 88 | 31.9% | 78 | 35.8% | 10 | 17.2% |  |
